# Performance of Sewer Concretes with Calcium Sulpho-Aluminate Cement and Portland Cement Blends: Field and Laboratory Studies

**DOI:** 10.3390/ma18061256

**Published:** 2025-03-12

**Authors:** Alice Titus Bakera, Amr Aboulela, Mark G. Alexander, Alexandra Bertron, Matthieu Peyre Lavigne, Samuel Meulenyzer, Cédric Patapy

**Affiliations:** 1 Department of Civil Engineering, University of Cape Town, Rondebosch 7701, South Africa; bakera.alice@udsm.ac.tz (A.T.B.); mark.alexander@uct.ac.za (M.G.A.); 2 College of Engineering and Technology, University of Dar es Salaam, Ubungo, Dar es Salaam 16103, Tanzania; 3 LMDC, Université de Toulouse, UPS, INSA, 31000 Toulouse, Francepatapy@insa-toulouse.fr (C.P.); 4 TBI, Université de Toulouse, CNRS, INRAE, INSA, 31000 Toulouse, France; mpeyrela@insa-toulouse.fr; 5 Holcim R&D, LCR, 95 Rue Montmurier, 38291 Saint Quentin Fallavier, France; samuel.meulenyzer@holcim.com

**Keywords:** biogenic acid deterioration, calcium sulpho-aluminate (CSA) cement, iron-based additive, sewer concrete, biogenic acid concrete (BAC) test

## Abstract

This paper discusses the performance of calcium sulpho-aluminate (CSA) cement and a Sulphate-Resisting Portland Cement (SRPC) with a fly ash (FA) additive (i.e., a SRPC + FA binder system) in a ‘live’ sewer environment; it deepens the understanding of their deterioration mechanisms by using a laboratory test for simulated sewer conditions. It also studies the role of an iron-based additive (‘Hard-Cem^®^’, HC) in improving the performance of SRPC + FA concrete under a biogenic acid attack. The performance of 0.4 w/b concrete specimens of the three binders (CSA, SRPC + FA, and SRPC + FA + HC) with calcite aggregates in sewer exposure was assessed by visual observation, measurements of mass and thickness changes, and microstructural analysis for approximately 25 months. The laboratory test, i.e., the Biogenic Acid Concrete (BAC) test, was used to study the deterioration mechanisms of these binders in terms of leaching solution pH and standardised cumulative leached calcium and aluminium. The results indicate that CSA concrete had improved performance in the sewer environment, showing no mass loss and only about one-third of thickness lost in the SRPC + FA concrete over a 25-month exposure period in the sewer environment. The BAC test results complemented the field observations. The iron-based additive in sewer concrete slightly reduced mass loss, likely due to its better resistance to abrasion and erosion, but not due to any chemical influence, since it does not participate in hydration or dissolution reactions. The findings imply that CSA cement may represent a suitable alternative binder for concrete sewer construction. They also suggest that a surface hardener has limited benefits, except when it is under abrasive conditions. Further investigation is required, especially since CSA contains high amounts of sulphate, the effect of which is not well understood.

## 1. Introduction

Globally, 70–95% of the urban population in high-income countries, and 10–40% in lower- and middle-income countries, respectively, are served by sewer systems [1]. These systems play a fundamental role in public health and environmental protection through managing municipal wastewater, which contains contaminants from domestic, industrial, and urban sources [2]. As most sewer infrastructure—including pipelines, outfalls, and manholes—is constructed from concrete and buried underground, it is often neglected, resulting in catastrophic failures, primarily due to inadequate maintenance and prolonged exposure to aggressive sewer conditions associated with biogenic acid corrosion.

Biogenic acid deterioration of sewer concrete is associated with sulfuric acid produced by microorganisms in sewers [3,4], and not purely chemical sulfuric acid attack, which significantly differs in its deterioration mechanisms [5,6,7]. In pure chemical sulfuric acid (H_2_SO_4_), without the involvement of microorganisms, aggressivity depends on the pH of the acid solution. In the case of biogenic acid attack, microorganisms, such as Sulphate-Reducing Bacteria (SRB), decompose organic and sulphate compounds to produce sulphides in wastewater [8,9]. Due to sewer hydraulic actions, these sulphides are released into the gas phase above the wastewater as hydrogen sulphide gas (H_2_S), which is then adsorbed on the unsubmerged concrete sewer wall [5,8,10]. Under a sufficient supply of oxygen (O_2_) and moisture, H_2_S is oxidised to sulfuric acid (H_2_SO_4_), abiotically and biotically. Under abiotic conditions, oxidation occurs chemically, while under biotic conditions, Sulphide-Oxidising Bacteria (SOB) are involved in the oxidation reaction [11]. The proliferation of SOBs on the sewer concrete surface occurs in two groups (neutrophilic (NSOB) and acidophilic (ASOB)), depending on the pH of the concrete surface [12,13]. The neutrophilic SOBs start colonising the surface by reducing the pH to <4, and then acidophilic SOBs take over by producing very aggressive sulfuric acid (H_2_SO_4_) [14,15]. The acid reacts with the cementitious hydrates and alkaline components of the concrete, leading to their dissolution and/or the formation of a deteriorated layer rich in gypsum (CaSO_4_·2H_2_O) with low strength, integrity, and pH [16,17,18].

The sewer concrete surface layer undergoes further deterioration due to continuous acid production, leading to the entire decomposition and neutralisation of all available hydrates, anhydrous phases, and reactive aggregates in the concrete. Typically, this degradation manifests as cracking, spalling, and material loss, which may eventually result in exposure and deterioration of reinforcing steel. Consequently, it may contribute to sewer leakage, collapse, sinkholes, and flooding, generating environmental pollution that adversely impacts public and ecological health. Such failures signify considerable direct expenses associated with sewer repair and rehabilitation, and indirect costs linked to the productivity losses of other functioning entities and social service interruptions due to the inoperative sewer [19,20,21].

In Australia, for instance, the comprehensive cost associated with repairing and rehabilitating sewer systems was estimated at AUD 69.7 billion during the fiscal year 2011–2012 [2]. Projections suggest this expenditure may rise to approximately AUD 125 billion annually over the subsequent two decades. Likewise, replacing 24,000 km of sewer infrastructure in New Zealand is anticipated to incur a cost of around NZD 15.8 billion, with an annual budget of NZD 17 million specifically allocated for sewer pipeline repairs [2]. The United States is similarly confronted with a considerable economic burden, as sewer rehabilitation costs were reported to be USD 3.3 billion US in 2009, whereas, in 2016–2025, about USD 532 billion were invested in water and wastewater infrastructure, with USD 134 billion spent in 2019 alone [22,23]. In Europe, similar trends can be observed; for example, about 20% of the sewer network in Germany requires short or mid-term rehabilitation, which accounts for approximately EUR 4 billion of annual investment in sewer rehabilitation [24]. In Belgium, the expense is estimated to be around 10% of the entire wastewater treatment cost [25,26]. The Netherlands annually spends about EUR 800 million on the rehabilitation and/or replacement of sewers, while France lacks about EUR 1.4 billion for each, based on its current expenditure [27]. These figures underscore the urgent need for effective corrosion mitigation strategies and sustained infrastructural investments to ensure the sustainability and resilience of sewer networks globally.

Given the significant financial and structural implications of biogenic acid deterioration, various mitigation techniques have been employed to address this problem, including sewer linings, protective coatings, and acid-resistant binders that prevent or slow the deterioration [28,29,30,31]. Linings are applied to avoid disruptions associated with sewer pipe excavation and replacement, particularly in cases where accessibility to the sewer line is limited. However, most liners are thermoplastic-based and are often expensive and less effective in certain situations, such as in uneven concrete surface profiles and the infiltration between the liner and host pipe [32,33]. Furthermore, using these liners can result in a substantial accumulation of aggressive gases within the sewer system, posing a threat to other unprotected components such as manholes. On the other hand, protective coatings such as epoxy coatings and antimicrobial polymer fibres or metal-zeolites can demonstrate commendable performance in laboratory settings; however, their efficacy can vary in real-world applications. For instance, epoxy coatings are known to delaminate, and antimicrobial agents dissolve in wastewater [31]. To overcome these problems, there is a need to explore and improve cement-based liners or produce acid-resistant concrete pipes manufactured using readily available and high-performance binder systems.

Calcium Aluminate Cement (CAC) has been widely used in cement-based liners of concrete sewer pipes, and occasionally as an acid-resistant binder [34]. Its superior performance is due to a long chain of dissolution, high neutralisation capacity of its hydrates, and gibbsite stability at a pH between 3 and 4 in the deteriorated layer [35]. However, CAC is expensive, especially compared to Portland Cement (PC), and therefore, it is mainly used in linings [36]. Thus, exploring the performance of other alternative binders, such as Calcium Sulpho-Aluminate cement (CSA), an aluminium-bearing binder that might present a cost-effective performance, is essential.

CSA represents a variant of CAC, first synthesised by Ragozina in 1957 and further developed by Alexander Klein in the 1960s [37]. It is produced by burning a blend of limestone, bauxite, and anhydrite or gypsum at a temperature between 1250 °C and 1300 °C, which is then blended with 15% to 25% of additional gypsum. It consists of three main phases: primarily ye’elimite ($C_{4}A_{3}\bar{S}$), minor belite (C_2_S), and gypsum [37]. The hydration of ye’elimite depends on the presence of gypsum and lime within the system. In pure water, it generates monosulfate and gibbsite; however, in the presence of gypsum, it produces ettringite, monosulphate, and gibbsite, with monosulfate predominantly forming when gypsum is depleted. When only lime is present, it results in the formation of a hydrogarnet phase and an AFm phase (monosulfoaluminate). Conversely, when both lime and gypsum are present, it yields ettringite, which is characterised by significant expansion [37,38]. Due to its low reactivity, the hydration of belite takes place at a later stage; it reacts with amorphous gibbsite (AH_3_) to form strätlingite (C_2_ASH_8_) rather than calcium silicate hydrates (C-S-H). Usually, ye’elimite and gypsum hydrations occur in the first several days, while belite may remain un-hydrated even after 90 days [39].

CSA concrete has been used for approximately four decades in China to manufacture self-stressing concrete sewer pipes. Nevertheless, there exists limited knowledge pertaining to its performance in sewer environments [40]. One related study is by Yang et al. [41], who studied the performance of artificial reef concrete made of CSA, sea sand, and seawater, compared to Ordinary Portland Cement (OPC) concrete with river sand and potable water under biogenic sulfuric acid in a marine environment. After subjecting the concrete specimens to a biogenic sulfuric acid simulator with *Acidithiobacillus ferrooxidans* bacteria for four months, it was observed that CSA outperformed OPC in terms of the degree of deterioration, mass loss, and compressive strength.

It is evident that the investigation of CSA cement performance within sewer environments is of paramount importance for the following reasons: (i) The quantity of Portlandite (CH) and calcium silicate hydrate (C-S-H) generated during the hydration process is minimal due to the lack of tricalcium silicate (C_3_S) and due to the presence of only limited amounts of dicalcium silicate (C_2_S) [42,43], thus reducing its susceptibility to acid attack, including biogenic acid attack. (ii) Its dominant hydration by-product is ettringite, which is unstable at a pH of less than 10.7 and is also known to be expansive, which might result in internal cracking and loss of strength in the long term [39]. (iii) Another hydration products is gibbsite, which contributes to resistance against biogenic acid attack in CAC systems, as it is stable at a pH of 4, which may also increase its potential to resist attack. (iv) The pH of its interstitial solution may be much lower than that of PC [42]. This last point might have a negative impact: the lower the pH, the higher the bacterial colonisation. The reported pore solution pH values of CSA mortars are highly variable, ranging from 8.5 to 13 [44,45].

In the present work, the performance of CSA cement in a field sewer and in a simulated laboratory test were studied, together with two other binder systems, i.e., a blend of Sulphate Resisting Portland Cement and Fly Ash (SRPC + FA) and the same blend with an iron-based additive (HC) (SRPC + FA + HC) (which was supplied by the cement manufacturer) in order to study its potential to enhance the acid resistance of sewer concrete. The partial replacement of SRPC with fly ash was deemed to improve the concrete microstructure by reducing its permeability [46]. Based on previous preliminary work at the University of Cape Town (UCT), the iron-based additive was observed to increase the resistance of concrete under biogenic acid attack, but without information to explain its performance. Also, in the literature, iron-attributed phases are alleged by some to reduce biogenic acid corrosion rate by providing a bacteriostatic effect and neutralisation capacity to the attacking acid [46]. Others [47,48], however, claim that they accelerate the attack by introducing internal microcracking in the intact concrete as a result of iron ion accumulation near the concrete deterioration layer. For these reasons, studying its performance in the SRPC + FA concrete was considered valuable.

Recently, Damion and Chaunsali [49] examined the performance of the same binder systems (in paste or mortar form) as in the present work, i.e., PC and CSA (with relatively high ye’elimite content), in both lab tests with concentrated acids, and in field conditions of a wastewater treatment plant. In terms of biogenic acid resistance, CSA outperformed PC, with the superior performance being attributed to the acid neutralisation capacity of the gibbsite phase. Bactericidal properties were also claimed, a factor that has previously been challenged by Buvignier et al. and Erbektas et al. [50,51]. The main factors of the biogenic acid resistance were indicated as the stability of the hydrated phases and the secondary phases formed from the biogenic acid attack, the neutralisation capacity, and the buffering effect of the binders [52]. The acid neutralisation capacity was shown to be higher for CSA compared to PC-based materials and lower than that of CAC [53].

This study had two objectives: the first and most important was to evaluate and compare the performances of CSA and SRPC + FA binder systems in a ‘live’ field sewer environment and a laboratory sewer-simulated test condition (BAC test [54]), and thereby, to deepen understanding of their deterioration mechanisms; and of secondary importance is to assess and understand the role of an iron-based additive in influencing the resistance of SRPC + FA concrete to biogenic acid attack. This additive was used in the experimental programme because there was an expectation that it could positively improve resistance under biogenic acid attack, based on the literature.

## 2. Materials and Methods

### 2.1. Materials

Cementitious materials used in this study were CEM I 52.5 N-SR3 (SRPC) and Calcium Sulfo-Aluminate-ye’elimite (CSA) cement. Fly ash (FA) was used as a supplementary cementitious material with the SRPC (hereafter also termed ‘SRPC-based binder’), while an iron-based additive (HC) was used as additional material. Holcim, France, supplied these materials, with the chemical compositions shown in Table 1. According to EN 197-1 [55], SRPC is a sulphate-resisting Portland cement with a C_3_A content of less than 3% (SR3). CSA cement used was Calumex^®^ Quick (CALTRA Nederland BV, Mijdrecht, The Netherlands). The mineralogical phases of anhydrous CSA are shown in Table 1. The FA was a pulverised fuel ash. The iron-based additive was Hard-Cem^®^ (HC, CTS Cement Manufacturing Corp., Cypress, CA, USA), consisting of iron particles.

**Figure 1:**
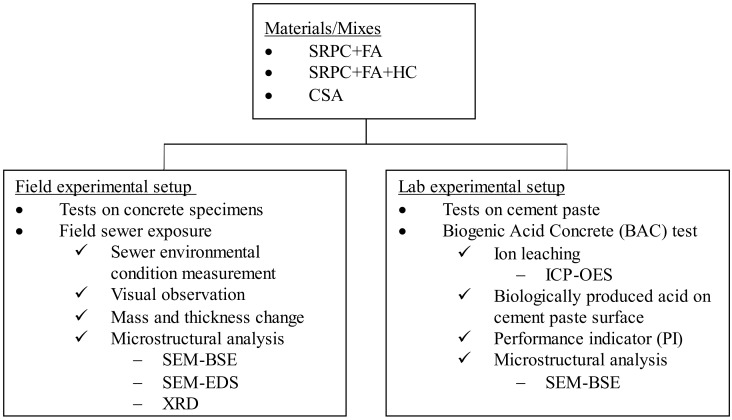
Methodology flow chart.

FA substituted 20% of SRPC to produce a blended binder system, which should perform better than plain concrete in sewers according to Kiliswa et al. [35]. Regarding HC, 11.4% of the total binder (20% FA and 80% SRPC) was added to study the influence of the iron-based additive component in concrete under biogenic acid attack. Crushed calcite coarse and fine aggregates and siliceous sand were used as aggregates. Figure 1 shows a flow chart of the methodology.

### 2.2. Field Experimental Setup

#### 2.2.1. Concrete Mixes and Casting Procedures

The concrete mixes shown in Table 2 were designed and cast by Holcim, France, for the live sewer exposure. Constituents of the concrete mix were kept constant, except for the binder type, to ensure that any corrosion effects were solely related to the binder. A low water content of 133 kg/m^3^ was used. The concrete mixes were ‘earth-dry’ and needed heavy mechanical compaction, similar to ‘dry’ mixes used in sewer pipe manufacture. A superplasticiser was used to enhance concrete castability.

Concrete specimens (150 mm cubes) were cast using vibration and compression techniques on account of the ‘earth-dry’ nature of the mixes. These were steam-cured for three days and then packed securely for dispatch to the UCT civil engineering laboratory (Cape Town, South Africa). After delivery, all concrete cubes were plastic-wrapped and stored in an environmental room at 21 ± 2 °C and 60% relative humidity. At 28 days of age, three concrete cubes per mix were tested for saturated density and compressive strength, according to SANS 5863 [56]. A further two cubes were used for durability index testing, viz. Oxygen Permeability Index (OPI) and Water Sorptivity Index (WSI) (SANS 3001-CO3-1 [57], SANS 3001-CO3-2 [58], see also, DI testing Procedure Manual [59]. The remaining cubes were prepared for sewer exposure; see Section 2.2.3.

The density of the SRPC + FA mix was similar to the theoretical value, see Table 2, but the SRPC + FA + HC and CSA densities were somewhat lower than the expected densities. This can be related to the lab-compaction of these concretes, due to the earth-dry nature of the mixes. In practice, they are compacted by heavy mechanical compaction in concrete pipe manufacture. Thus, there were some internal compaction voids. Consequently, their compressive strengths at 28 days were somewhat depressed. However, all OPI values were above 10, indicating good quality concrete (based on the criteria in Alexander et al. [60]), with low pore-interconnectivity. WSI values also indicated excellent to good quality concrete. The water-penetrable porosities were somewhat higher than comparable values for sewer concretes, typically 4–6%, in relation to the compaction voids. Notwithstanding this, the quality of the concrete cubes was deemed acceptable for the sewer exposure study since the main deterioration mode would be bio-chemical.

OPC mortar was systematically used in every campaign (except for campaign III, which was not taken into account for the PI calculations) as a reference material. The reference material was always prepared at LMDC using the same cement. However, the rest of the cementitious materials were prepared either by partners of the different projects or at LMDC.

#### 2.2.2. Sewer Site Background and Environmental Conditions

**Figure 2:**
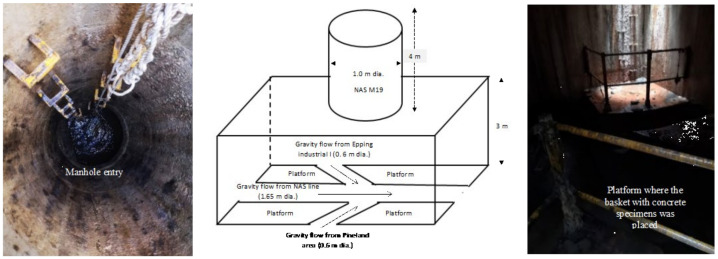
Schematic of the NAS M19 showing its interior, wastewater collecting channels, and the concrete platform on which the baskets with concrete specimens for this study were positioned.

The overall site comprises a substantial outfall sewer with a diameter of 1000 mm, the so-called Northern Area Sewer (NAS), extending over a total length of 8.7 km, in the City of Cape Town, South Africa, and serving an area of approximately 4000 ha. Manhole 19 (NAS MH 19) was used for the exposure of the specimens based on the H_2_S gas concentrations—see Section 3.1.1. Figure 2 shows details of the NAS MH19 site.

The sewer environmental condition analysis involved measuring sewer headspace gas concentrations of H_2_S and carbon dioxide gas (CO_2_), temperature, and RH for 8 to 12 h/day, once a month for a year.

#### 2.2.3. Concrete Specimen Preparation for Sewer Exposure

**Figure 3:**
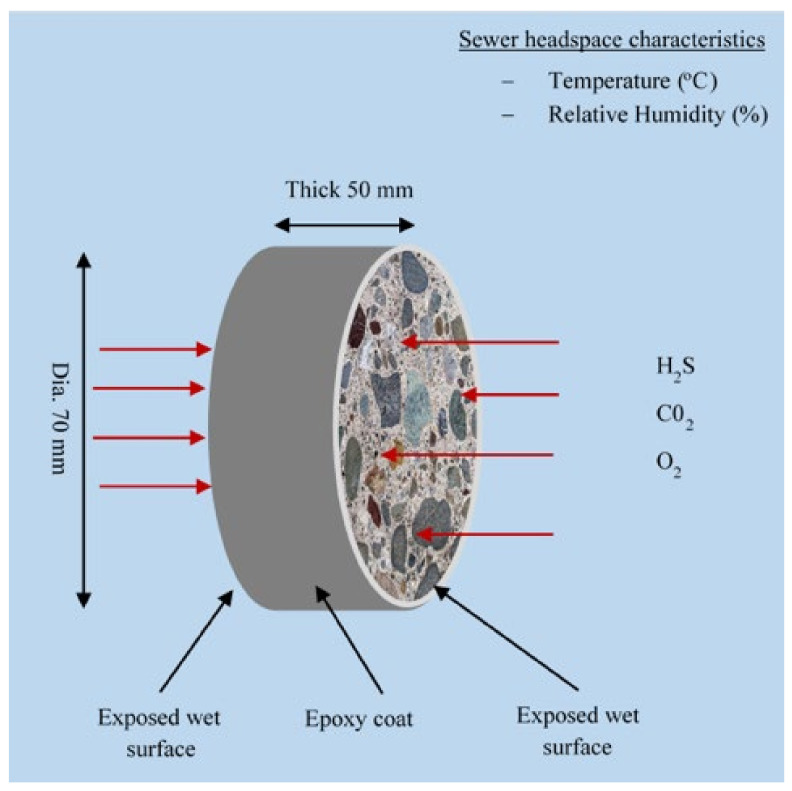
Concrete specimen setup for site exposure showing exposed surfaces.

Concrete cubes for sewer exposure were cored, epoxy-coated, and then cut to obtain concrete discs of 70 mm diameter and 50 mm thickness. The cylindrical surface of the specimens was epoxy-coated to allow uni-axial exposure on two opposite nominally identical cut sections, see Figure 3. The epoxy coating was applied in three layers on a sandpapered, cleaned, and dried surface. For each mix, five concrete discs were prepared.

#### 2.2.4. Concrete Deterioration Monitoring Strategy

**Figure 4:**
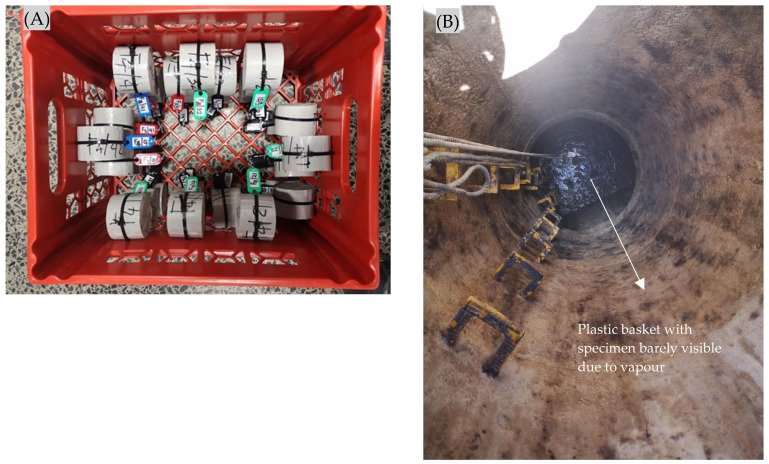
Concrete specimens fixed in the plastic basket for site exposure (**A**). View from the top (**B**), the manhole shaft, showing a rope used to lower the basket to the manhole concrete platform described in Figure 2.

The initial mass, dimensions, and surface pH of the concrete discs were measured before exposure, followed by immersion in tap water for three days to achieve saturation. After saturation, their masses and dimensions were again recorded, and the specimens were fixed in a plastic basket for exposure, see Figure 4. The specimens were orientated in the basket with vertical circular (exposed) faces. The exposure environment was primarily gaseous, i.e., H_2_S and other gases, which could flow freely around the specimens. The basket was located on the site (NAS M19) for exposure. A 10 m rope was secured to lower the basket through the manhole shaft until it rested on the concrete platform illustrated in Figure 2. After two months of exposure, the basket containing specimens was retrieved from the manhole, placed in a plastic bag to minimise evaporation, and transported to the laboratory for routine measurements and observations, including visual assessments, mass measurements, and thickness evaluations. The thickness measurements were conducted at six positions (permanently marked for subsequent monitoring periods) around the circumference using a vernier calliper with a precision of 0.02 mm. A precision balance with a capacity of 3000 g and a sensitivity of 0.01 g was used for mass measurement. This process was performed on the same day, and the specimens were immediately re-secured in the basket and returned to the site for continued exposure. The monitoring campaign was repeatedly regularly, varying from monthly to quarterly for 25 months. At the end of the exposure period, a corroded sample was cut for the microstructural analysis from each mix.

#### 2.2.5. Microstructural Analysis on Concrete Specimens Exposed on Site

Microstructural analyses were carried out using scanning electron microscopy (SEM) and X-ray diffraction (XRD) techniques to evaluate the impact of biogenic acid exposure on the concrete microstructure. In order to maintain the integrity of the corroded surface, the specimens underwent a gentle brushing to eliminate any organic material present, after which they were immersed in an isopropanol solution in a desiccator for seven days to displace free water. Subsequently, the specimens were subjected to drying in an oven at 40 °C for three days. Following this, each specimen was bisected: one half was allocated for SEM analysis, while the other half was designated for XRD analysis.

The specimen for SEM analysis was epoxy-impregnated to protect the microstructure. It was cut to obtain a 20 × 20 × 10 mm piece, which was mounted in a mould with the studied surface, i.e., a 20 × 20 mm cross section, facing down. The specimen in the mould was again epoxy-impregnated, followed by surface polishing as recommended in Scrivener et al. [61] and ASTM C 1723 [62]. It was then thinly coated with carbon to avoid surface charging before inserting the polished sample into the SEM vacuum chamber. The analysis was conducted using an FEI Nova NanoSEM 230 (FEI, Hillsboro, OR, USA) at the UCT Centre for Imaging and Analysis to obtain BSE images and elemental transverse point analysis into the concrete depth. BSE images were collected by a BSE detector in low vacuum mode with an accelerating voltage of 20 kV and beam intensity of 10. For elemental transverse point analysis, a total of five BSE images of 400 µm view field (to give 2 mm depth from the concrete exposed surface), each image with 40 spot points at 10 µm distance apart, were analysed using the Oxford EBSD system with Aztec HKL software (https://nano.oxinst.com/products/aztec/aztechkl, accessed on 1 January 2022).

XRD analysis was conducted on flat surfaces of the intact concrete and deteriorated samples. The deteriorated sample was analysed in layers, starting with the exposed surface, i.e., 0 mm, and moving deeper into the concrete. After analysing the exposed surface, the surface was further polished using P800 sandpaper (3M, Maplewood, MN, USA) to create new layers, i.e., at 100 µm and 200 µm depth from the exposed surface. The procedure was repeated until soft, and the degraded deposits had been removed. In the intact concrete, only one layer at about 5 mm from the exposure surface was analysed. The data were collected using a Brucker D8 diffractometer (Billerica, MA, USA, Ver. Eva 4.1.1) in the θ-θ configuration using a monochromator incident beam and CuKα radiation (λ = 1.54Å) with a rotating sample holder. The mineralogical phases were identified using the EVA software.

### 2.3. Laboratory Experimental Setup

#### 2.3.1. Biogenic Acid Concrete (BAC) Test

The Biogenic Acid Concrete (BAC) test, a laboratory sewer-simulated condition, was developed at INSA (National Institute of Applied Sciences) in Toulouse in France to evaluate the resistance of cementitious-based materials to biogenic sulfuric acid environments in a lab setup. The test was used to deepen the understanding of the deterioration mechanisms of the three binder systems (SRPC + FA, SRPC + FA + HC, and CSA).

**Figure 5:**
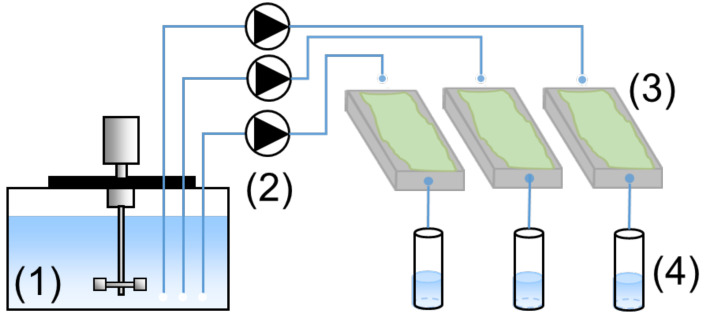
Schematic diagram of the Biogenic Acid Concrete (BAC) test [41].

Figure 5 shows a schematic diagram of the BAC-test experimental pilot design [63]. The test pilot consisted of a temperature-controlled 200-litre tank (4 °C) containing feeding solution for the microbial growth (1) linked to the exposed samples by flow pumps connected by ptfe and silicon tubes (2), which deposit the feeding solution in a controlled flowrate on the specimens (3). The surfaces of the specimens were seeded with microbial inoculum obtained from activated sludge containing SOBs, which convert reduced sulphur into sulfuric acid on the specimen’s exposed surface. The specimens were laid on moderately inclined supports to induce the flow of the liquid and optimise the collection of the leaching solution in contact with the cementitious material.

Tetrathionate salts (K_2_S_4_O_6_) were used as a soluble reduced sulphur source in the feeding solution, mixed with other nutrients, mainly nitrogen, phosphorus, iron, and oligo-elements, required for microorganism growth. The feeding solution was prepared with de-ionised water to avoid contamination by calcium, magnesium, and other cations. The leaching solutions were regularly collected (two samples per week) (4) and filtered at 0.45 μm to exclude microorganisms in the liquid samples. The liquid samples were then stored at 4 °C after measuring the pH for the chemical analysis. The representativeness and reproducibility of this test method have been demonstrated in previous studies [54,63,64,65].

#### 2.3.2. Cement Paste Preparation for Exposure to the BAC Test

Cement paste specimens for the BAC test were prepared at a w/b of 0.4 according to EN 197-1 [55]. The specimens were demoulded 24 h after casting, immediately plastic wrapped, and cured at 20 °C and 50% relative humidity (RH) for at least 28 days before being subjected to the BAC test.

The cement paste specimens exposed to the BAC test were 20 × 40 × 80 mm prisms obtained from sawing 40 × 40 × 160 mm mortar specimens into 4 equal parts. An epoxy resin—EUROKOTE 48-20—was applied on the mortar specimen surfaces, leaving only the top surface exposed to the biodeterioration test, see [63]. Two thin lines (1 mm) of resin were also applied to the edges of the exposed surface to contain the solution flow from the upstream to the downstream side, and to preserve a reference of the initial thickness for further observations.

#### 2.3.3. Cement Paste Deterioration Monitoring Strategy

The main oxides from cement paste considered in this study are CaO, SiO_2_, and Al_2_O_3_, with calcium (Ca^2+^), aluminium (Al^3+^), and silicon (Si^4+^) ions as the associated cations after acid attack. During the attack, the Ca^2+^ ion is the most readily leached from the matrix, followed by Al^3+^, and then Si^4+^. The reactivity of the different binder systems in this study was compared after quantifying Ca^2+^ and Al^3+^ in the leaching solution, collected downstream of each specimen and standardised by the initial total Ca^2+^ and Al^3+^ contained in the specimens.

Based on these analyses, a performance indicator (PI) was defined, combined with post-treatment calculations detailed in Section 2.3.4, Section 2.3.5 and Section 2.3.6. This PI, validated on CAC and PC [66], was used to classify materials according to their resistance to biogenic sulfuric acid attack in sewer conditions.

#### 2.3.4. Analyses of the Leached Solutions

The concentrations of the cementitious cations, i.e, Ca^2+^, Al^3+^, and Fe^3+^, in the leached solutions from the BAC test were determined using Inductively Coupled Plasma with Optical Emission Spectrometry (ICP-OES), using an Optima 7000 DV (Perkinelmer, Waltham, MA, USA), with a Meinhart nebuliser.

The sulphate (SO_4_^2−^) content of the leached solution from the biological oxidation, and potentially from the dissolution of the reactive phases of the exposed materials, were determined using Ion Chromatography (Dionex ICS-3000, Thermo Fisher Scientific, Sunnyvale, CA, USA).

The concentration of the tetrathionate (S_4_O_6_^2−^) in the leached solution was determined using high-performance liquid chromatography (Ultimate 3000) with an ultraviolet (UV) detector. A suitable flow rate of the feeding solution was used, with the carrier phase being injected at 0.6 mL/min and 23 °C. This phase was a solvent with a constant composition of water-acetonitrile (75:25 vol.%) and 15 mM of tetrapropylammonium hydroxide (TPA) [67]. Acetic acid was added to adjust the pH of the carrier phase to 5.0.

#### 2.3.5. Calculation of the Biogenically Produced Acid on Cement Pastes Exposed to the BAC Test

As mentioned earlier, sulphate in the leached solution comes from the biological oxidation of the tetrathionate and the dissolution of the sulphate-bearing reactive phases of the cementitious matrix, such as ettringite for the CSA. Based on Aboulela [66], deducting the concentration of S_4_O_6_^2−^ in the feeding solution from that measured in the leached solution provides the quantity of S_4_O_6_^2−^ oxidised biologically. Equation (1) calculates the amount of sulphate biologically produced.

S_4_O_6_^2−^ + 3.5O_2_ + 3H_2_O → 4SO_4_^2−^ + 6H^+^ (1)

Comparing the SO_4_^2^ concentration measured in the leached solution to the theoretical and biologically produced SO_4_^2−^, the amount of SO_4_^2−^ released from the different materials was estimated. This approach has been validated on different cementitious materials with different chemical and mineralogical compositions. In this context, it was shown that the amount of acid produced does not depend directly on the nature of the cementitious material [66].

#### 2.3.6. BAC Test Performance Indicator (PI)

The analyses of leached solutions provided the concentration of Ca^2+^ and Al^3+^ with time. Thus, the flux of leached calcium for each specimen at day d_n_ could be calculated using Equation (2).

F(Ca^2+^)_dn_ = Q_dn_ × [Ca^2+^]_dn_ × 10^−3^, (2)

with F(Ca^2+^)_dn_: flux of leached calcium ions at day d_n_ (in mol/h); Q_dn_: flow of leached solution at day d_n_ (in mL/h); [Ca^2+^]_dn_: concentration of leached calcium measured in the leached solution at day d_n_ (in mol/L).

The same calculation was carried out at day d_n+1_ to obtain F(Ca^2+^)_dn+1_ using the amount of calcium leached between day dn and day d_n+1_, calculated from Equation (3).

Tot(Ca^2+^)_dn+1−dn_ = ((F(Ca^2+^)_dn+1_ + F(Ca^2+^)_dn_)/2) × (d_n+1_ − d_n_), (3)

with Tot(Ca^2+^)_dn+1−dn_: cumulative leached calcium between day d_n+1_ and day d_n_, expressed in mole; (d_n__+__1_ − d_n_): the time between day d_n+1_ and day d_n_, expressed in days.

The same calculation was performed for Al^3+^. Each day, the total leached Ca^2+^ and Al^3+^ sum was expressed as “Cumulative Ca + Al Leached”. The “Cumulative Ca + Al Leached” was then standardised by the initial amount of Ca^2+^ and Al^3+^ in the exposed material (in mol) at each sampling day to express a performance indicator “PI”, calculated using Equation (4) as a percentage.

PI_dn+1−dn_ = [(Tot(Ca^2+^)_dn+1−dn_ + Tot(Al^3+^)_dn+1−dn_)/(initial Ca + initial Al)] × 100. (4)

#### 2.3.7. Cement Paste Microstructural Analysis

At the end of the BAC test, microstructural and chemical analyses were carried out on the cement paste specimens using Scanning Electron Microscopy (JEOL JSM-6380LV with accelerating voltage 15 Kv, Akishima, Japan) in backscatter electron (BSE) mode, also with point analyses using energy dispersive spectroscopy EDS (Rontec XFLASH 3001, Berlin, Germany). The combination of SEM and EDS results was used to study the impact of the iron-based additive on the evolution of the chemical composition of the hydrated cement paste. These results are presented in Section 4.2.

## 3. Results and Discussion

### 3.1. Field Sewer Conditions

Results for the field sewer conditions are presented in six subsections: sewer environmental conditions after up to one year; visual observations, mass and thickness changes, and XRD analyses after up to 25 months of exposure; and concrete microstructure observations (SEM-BSE) and elemental profiles (SEM-EDS) in the deteriorated concrete specimens after up to 9.6 months of exposure. This is because obtaining clear BSE images for EDS analysis at 25 months of exposure was complex due to the high proportion of aggregate particles in the concrete microstructure.

#### 3.1.1. Sewer Environmental Condition

Gas concentrations (H_2_S and CO_2_), temperature, and relative humidity for the NAS MH 19 site were measured in the four seasons of the year 2021, i.e., summer (November to February), autumn (March to April), winter (May to August), and spring (September to October).

Throughout the year, the sewer headspace parameters, particularly gas concentrations, exhibited both diurnal and seasonal fluctuations. The highest H_2_S concentrations were recorded in March at 104.6 ppm and April at 101.1 ppm. In contrast, the lowest concentrations were observed in September, measuring 11.5 ppm. Seasonal averages for the H_2_S concentration were as follows: autumn 23.6 ± 9.3 ppm, winter 6.4 ± 2.0 ppm, spring 6.8 ± 3.2 ppm, and summer 13.6 ± 8.5 ppm. On average, the CO_2_ concentration was 9000 ± 1000 ppm, while the winter, spring, and summer values were 28,000 ± 5000 ppm, 48,000 ± 2000 ppm, and 43,000 ± 9000 ppm, respectively. Consequently, the specimens exposed to these varying conditions did not experience constant gas environments, unlike those subjected to controlled laboratory conditions [68,69,70].

The sewer headspace exhibits considerable temperature fluctuations throughout the year, ranging from 15 °C in winter to 30 °C in summer, which influenced the sewers’ biological and chemical processes. In winter, persistently low temperatures can significantly inhibit microbial activity and slow the decomposition rates of organic materials. Conversely, in summer, elevated temperatures enhance microbial processes and accelerate the breakdown of organic matter, resulting in increased H_2_S and CO_2_ gas concentration. The RH levels in the sewer headspace exhibit an inverse trend to temperature. The highest values, as high as 100%, were typically recorded during winter, and the lowest of approximately 70% in summer. These humidity variations indicate a dynamic system where excess moisture encourages microbial growth, crucial for concrete deterioration. [14,15,48,71].

#### 3.1.2. Visual Observations

**Figure 6:**
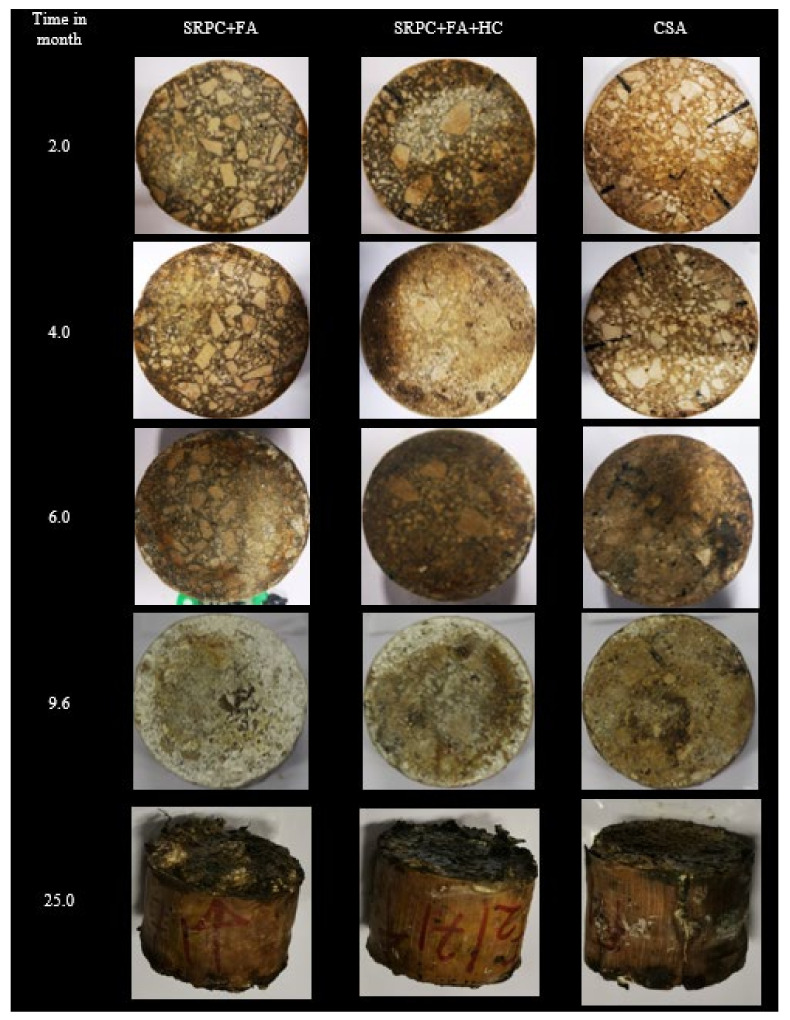
Visual observation of concrete specimens exposed at NAS MH 19; different ages.

Figure 6 illustrates the evolution of the exposed concrete surfaces of the three binder systems over time. This evolution occurs as microorganisms proliferate and produce acids interacting with the concrete constituents, leading to deterioration. The images captured prior to exposure were distorted; however, no significant changes were observed following 2 months of exposure. The concrete specimens began exhibiting signs of deterioration after approximately 6 months of exposure, with increasingly apparent deterioration and deposits noted after 9.6 months. The exposed surfaces exhibited slight discolouration after 2 months of exposure, which we attributed to wastewater contamination and the presence of a microorganism biofilm.

At 4 months of exposure, crystalline-type products with ‘granular’ particles were observed on the surface of the concrete, being the initial deterioration products. After six months, clear signs of deterioration occurred, with minor deterioration deposits (white, powder-like) and reddish-brown staining on the concrete edges. After 9.6 months, intensive deterioration occurred, starting from the edge toward the centre of the specimens. The reddish-brown stains served to demarcate between corroded and non-corroded surfaces. Such staining was also observed in Grengg et al. [72]. After 25 months, the entire concrete specimen experienced massive deterioration both on the exposed and epoxy-coated surfaces. For the SRPC-based concretes, the exposed surfaces were covered with massive deterioration deposits, while for the CSA concrete, the most significant observation was the blisters and cracks on the epoxy-coated surface. This indicated concrete expansion, due to either ongoing ettringite production from hydration or acid penetration through the concrete–epoxy interface to form biodeterioration products.

The more pronounced degradation observed at the concrete edges, specifically at the interfaces between the concrete and the epoxy coating, can be attributed to the orientation of the specimens within the exposure baskets. The exposed surfaces were positioned vertically, facilitating the flow of acid over the surface towards the edges, resulting in accumulation at the epoxy coating. Consequently, the deterioration front across the exposed surfaces exhibited non-uniformity, as also documented in references [12,47].

Generally, the SRPC + FA concrete showed significant deposits of deterioration products, followed by SRPC + FA + HC concrete, with CSA concrete showing fewer deterioration products on the exposed surfaces, but with the epoxy coating damaged due to the expansion of the specimens. The visual observations provided a qualitative assessment of concrete deterioration performance.

#### 3.1.3. Mass and Thickness Changes

**Figure 7:**
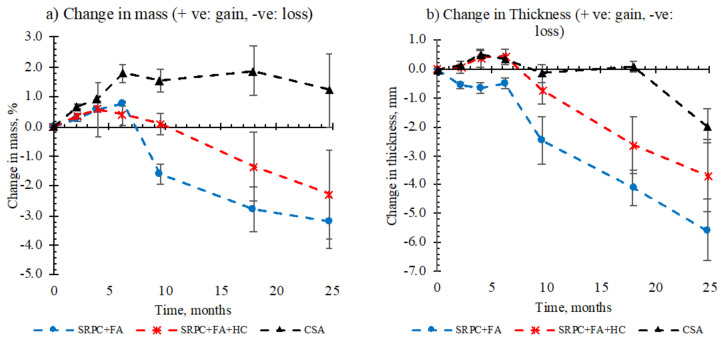
(**a**) Change in mass and (**b**) change in thickness for concrete specimens exposed at NAS MH 19 for 25 months.

Figure 7a shows mass change profiles of the concrete mixes exposed at NAS MH 19 for 25 months, expressed as percentage change in the original measurement before exposure. For the SRPC concretes, the profiles show mass gain up to about six months, followed by mass loss. CSA concrete, however, shows continuous mass gain for about 18 months, and then a slight mass loss thereafter. The mass change rate in any time interval differs among the concretes, with SRPC +FA generally showing a high rate, followed by SRPC+ FA + HC, then CSA.

The mass gain is associated with initial moisture absorption for SRPC concretes, and initial moisture absorption and deterioration products formed as blisters under the epoxy layer for CSA concrete. CSA cement requires a higher water/binder ratio than PC to achieve full hydration [73]; and consequently, since the CSA concrete had a low water/binder ratio of 0.4, the moisture absorbed could promote further hydration. On the other hand, the penetration of acid through the surface influences the formation of initial deterioration products that appeared as blisters on the epoxy coated surface. Eventually, the epoxy coating cracked at 25 months of exposure, see Figure 6. The initial deterioration products would be expected to be ettringite above pH 10.7, or gypsum following ettringite transformation below pH 10.7 [74].

Mass loss is mainly associated with material loss due to deterioration. CSA showed an insignificant mass loss, followed by SRPC + FA + HC concrete and then SRPC + FA concrete. This indicates that iron-based additives may have provided some additional resistance against physical abrasion which occurred occasionally in the sewer.

Due to the uneven and non-uniform deterioration over the exposed surface (Section 3.1.2), the thickness measurement in Figure 7b varied around the specimen perimeter; the error bars indicate the variability. Thickness reductions over 25 months of exposure were 5.56 ± 1.05 mm for SRPC + FA, 4.48 ± 0.27 mm for SRPC + FA + HC, and 1.97 ± 0.59 mm for CSA. After 19 months, the slope of thickness change in CSA is similar to or even higher than the Portland-based concretes, suggesting a delay in thickness loss. However, it was difficult to accurately assess thickness change in a relatively short period.

As with mass change, SRPC + FA experienced greater thickness loss than the other mixes. The improved performance of the SRPC + FA + HC concrete is, at this stage, ascribed to the surface hardness effect of the iron-based additive. Broadly, the thickness changes correlated with the mass changes. However, for the CSA concrete, while there was no change in thickness up to about 18 months, there was a substantial increase in mass. This is related to the continued formation of hydration products due to moisture absorption, and to deterioration products due to acid penetration into the concrete, leading to a mass increase, which can be accommodated in the available voids and pores.

#### 3.1.4. Concrete Microstructure Observations—SEM-BSE Images

**Figure 8:**
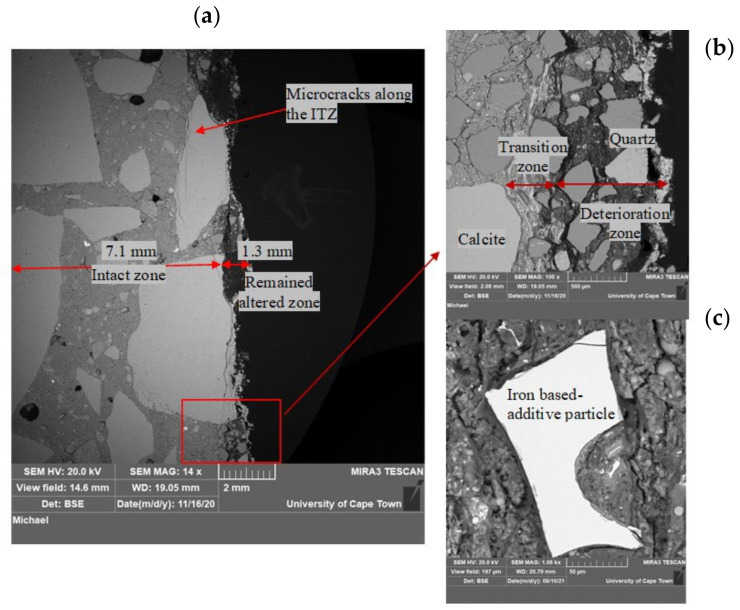
(**a**,**b**) show SRPC + FA BSE images after 9.6 months of exposure in the NAS M19. (**c**) shows the iron-based additive in the deteriorated zone of SRPC + FA + HC concrete; for more details, see the Appendix A, Figure A1.

**Figure 9:**
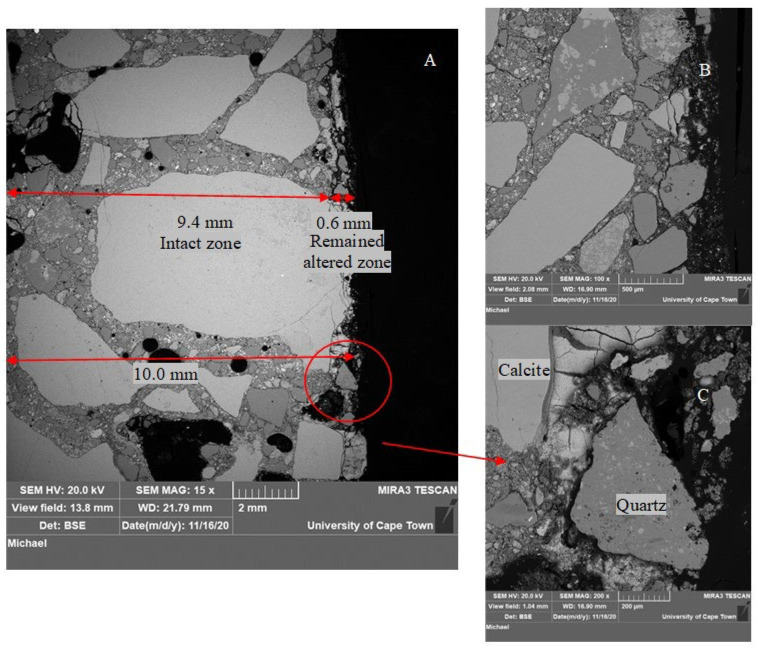
CSA BSE image after 9.6 months of exposure in the NAS M19. (**A**) shows altered zone about 0.6 mm wide; (**B**) shows cementitious matrix; (**C**) shows altered and intact zones.

SEM-BSE images on SRPC and CSA concrete samples after exposure to the sewer environment for 9.6 months are presented in Figure 8 and Figure 9. In all cases, two distinct zones are observed: an intact zone and an altered zone, which itself comprises an outer deterioration zone and an inner transition zone. The deterioration zone presented here is the remaining zone after thickness loss. The transition zone is a thin layer (<400 µm), which is not always clearly visible in the images, residing between the intact and deterioration zones.

In Figure 8a, the intact zone in the SRPC + FA mix had variable width depending on the aggregate particle position and the definition of the extremity of the altered zone. The altered zone also had a highly variable width, and in the present case, was about 1.3 mm wide, consisting of loose fine aggregate particles and a fragmented cement matrix with high porosity, with a grey level slightly different from the intact concrete. Microcracking through coarse aggregate particles and along the ITZ (Interfacial Transition Zone) was also observed. This microcracking is related to the coupled effect of chemical attack and sample preparation, ultimately leading to loss of cementitious matrix cohesion and aggregate detachment. On the exposed surface, coarse aggregate particles (calcite, which dissolves in acid solution) experienced uniform frontal dissolution. For this aggregate and under these sewer conditions, the acid reacts uniformly with the exposed aggregate surface without leaving any residue [75]. As a result, the aggregates lose their integrity and cohesion, becoming ‘soft’ deterioration products. In Figure 8b, the fine aggregates, i.e., quartz, detach from the cementitious matrix but remain unaltered, leaving the altered zone with whitish deposits, which are evidence of the conversion of cementitious products leading to cement–aggregate bond loss. This bond loss leads to increased porosity and the loss of matrix cohesion.

**Figure A1:**
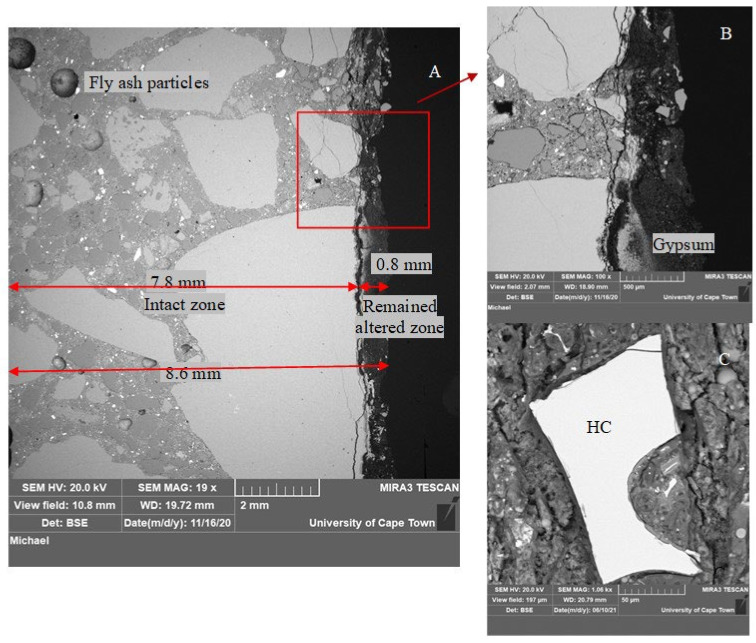
SRPC + FA + HC BSE image after 9.6 months of exposure in the NAS M19. (**A**–**C**) See also Figure 8 in main text.

Similar observations as those for SRPC + FA concrete were observed in the altered zone of the SRPC + FA + HC concrete (its BSE images are given in the Appendix A, Figure A1). Iron-based additives manifest as bright particles distributed throughout the concrete microstructure because the iron has high atomic weight. These particles appear to be not hydrating in the intact concrete or dissolving in the altered zone, indicating that they are not participating in chemical reactions in concrete, see Figure 8c.

CSA concrete, by contrast, has a different microstructure due to ettringite being its main hydration product. Figure 9A shows an altered zone about 0.6 mm wide, which is considerably less than the SRPC-based concretes. The altered zone differs from the intact zone by increased porosity and loss of cohesion. The cementitious particles are disaggregated in the altered zone, while they remain compact in the intact concrete. The brightness of both zones is similar, which makes it difficult to clearly demarcate between the altered zone (i.e., deteriorated and transition zones) and the intact zone, especially in the cementitious matrix, see Figure 9B. This is possibly due to a superposition of ettringite, i.e., the original ettringite from hydration, and the newly formed ettringite after sulphate penetration into the altered zone. In the transition zone, the penetration of further SO_4_^2−^ ions from the surface, together with the aluminate phases present, can form additional ettringite, at a pH > 10.7 [17]. This was shown in the reactive transport modelling (HYTEC Model) presented in [76]. However, the two zones can easily be distinguished in the aggregate matrix (see Figure 9C). As before, calcite aggregates dissolve and crack at the surface, while quartz remains unaltered. Concerning these microcracks in the altered zone, sewer pipes are usually designed with an internal sacrificial layer where deterioration occurs, leaving the structural barrel intact to resist imposed loads. Consequently, this cracking is not expected to affect structural pipe stability, but will affect long-term durability, assessed in the field study by mass and thickness changes.

As mentioned in Section 3.1.3, CSA concrete experienced insignificant thickness change until 19 months of exposure, but significant mass gain. This is related to the increasing formation of ettringite phases in the pore space and voids due to moisture absorption and sulphate ion penetration. In CSA concrete, anhydrous cement remains in the system after initial hydration, which can be activated for further hydration with a sufficient supply of moisture from the sewer, and the presence of alumina-bearing phases can promote the formation of secondary ettringite when they react with penetrating sulphate ions. The voids and porosity compensated for the increased volume by providing space for ettringite formation without causing substantial expansion of the concrete matrix.

#### 3.1.5. Profiles of Elements in the Deteriorated Concrete—SEM-EDS Analysis

The elements of interest were calcium (Ca^2+^), silicon (Si^4+^), sulphur (S^2−^), aluminium (Al^3+^), iron (Fe^3+^), and magnesium (Mg^2+^). Their profiles show the evolution of the cementitious matrix under acid attack [52]. The analysis concentrated on the cementitious matrix rather than the aggregate particles. However, it was impossible to entirely avoid aggregate particles in the analysis, hence the saw-tooth appearance of the profiles. The analysis was performed on a 2 mm depth of concrete from the deteriorated surface to observe the distribution of elements and how they characterise concrete zonation during the attack.

**Figure 10:**
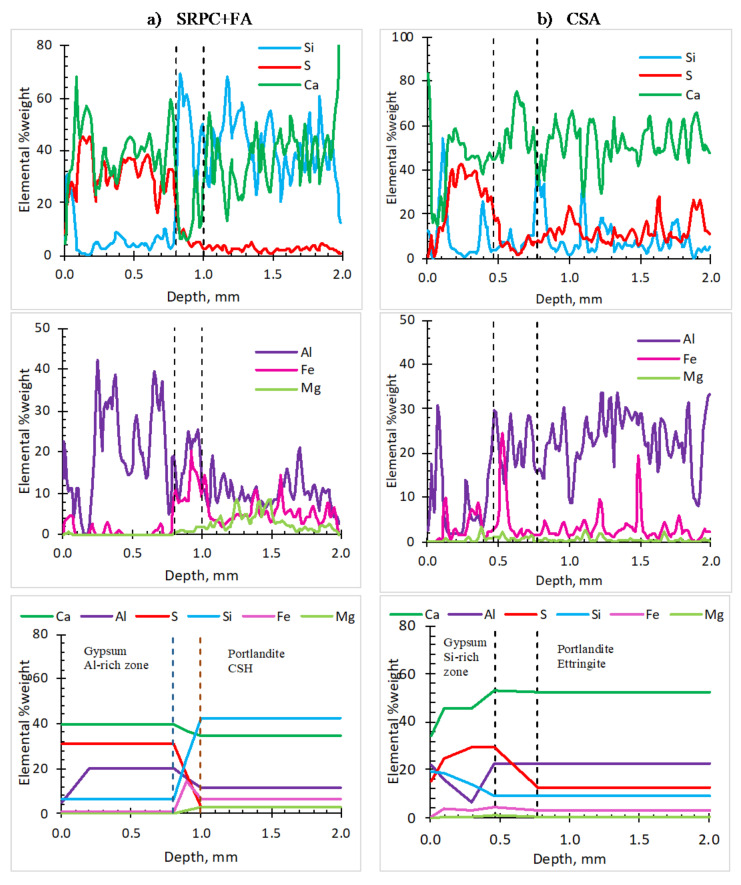
SEM-EDS elemental analysis on a 2 mm depth of (**a**) SRPC+ FA and (**b**) CSA concrete samples from the exposed surface; calcium (Ca); silicon (Si); sulphur (S); aluminium (Al); iron (Fe); and magnesium (Mg). Three zones, i.e., deterioration, transition, and intact zone, are shown inwards from the left-hand axis. The third row provides a schematic summary of the elemental distributions and postulated cement phases in the concrete zones.

Figure 10a (first two rows) shows a 2 mm depth of the SRPC + FA concrete from the deteriorated surface, with three zones—the deterioration zone and transition zone (collectively termed the altered zone earlier) and the intact zone—differentiated by a marked change in element concentrations. The deterioration zone (approximate thickness of 0.8 mm) is characterised by high sulphur, calcium, and aluminium concentrations, consistent with substantial gypsum and ettringite formation [48]. The transition zone is characterised by low calcium, a slight increase of silicon, and higher iron concentration, which is also observed by Jiang et al. [47] and Grengg et al. [48]. The intact zone has a high concentration of calcium and silicon but less aluminium and sulphur, consistent with the primary binders. The iron detected in the microstructure is mainly from fly ash and SRPC. Its concentration is slightly higher in the transition zone, while magnesium is generally of low concentration, especially in the deterioration zone, indicating the leaching of this element.

The SEM-EDS analysis of the SRPC + FA + HC concrete is shown in Figure A3 with similar elemental distribution in the zones as for SRPC + FA concrete. A higher iron concentration was expected throughout the profile due to the iron-based additive, but this was not observed. Consequently, the elemental profile per se could not shed light on the better performance of the SRPC + FA + HC concrete than SRPC +FA concrete. Its performance is rather attributed to the increased surface hardness and abrasion resistance of the concrete rather than any chemical contribution [72].

Figure 10b (first two rows) shows the elemental distribution in CSA concrete. A high concentration of ettringite is the main characteristic of this concrete, with calcium, sulphur, and aluminium observed throughout the depth. As expected, the silicon concentration was less in the intact zone compared to the SRPC concretes. The concentration of sulphur was highest in the deterioration zone and least in the transition zone. The concentration of aluminium, in contrast, was higher in the intact zone than in the deterioration zone. This likely indicated the transformation of ettringite to gypsum and aluminium-bearing phases in the deterioration zone [74]. Again, iron was mainly observed in the transition zone, while magnesium was insignificant.

The third row of Figure 10 summarises the elemental distributions and postulated cement phases in the concrete zones. The analysis indicates that sulphur is a key element in characterising biogenic acid deterioration in sewer concretes, deriving from the sulfuric acid that reacts with the concrete matrix to form gypsum and ettringite. Observing sulphur variation in detail can assist in determining the thickness of the deterioration zone that remains after thickness loss, which cannot be easily observed through physical/mechanical measurement or visual observation. On the other hand, the change in silicon and iron concentrations was used to define the transition zone in the two SRPC-based concretes, while for CSA concrete, the transition zone was defined by a slight decrease in sulphur concentration, followed by an increase in the deterioration zone.

In summary, a high concentration of simultaneous calcium and silicon implies calcium silicate phases (C-S-H) and portlandite, which are observed in the intact zone of the SRPC-based concretes. A combined high concentration of calcium, aluminium, and sulphur indicates the presence of ettringite as observed in the intact zone of the CSA concrete. A high concentration of calcium and sulphur indicates a strong presence of gypsum, observed in the deterioration zones of both CSA and SRPC-based concretes. The poorer performance of SRPC concrete is associated with the decalcification of C-S-H and portlandite because of their susceptibility to acid attack. The better performance of CSA may be associated with ettringite formation due to sulphate ion penetration in the transition zone, which increases the neutralisation potential of the matrix at pH >10.7 and, later, transforms to form gypsum and aluminium-bearing phases in the deterioration zone where the pH is below 10.7.

#### 3.1.6. XRD Analyses

**Figure 11:**
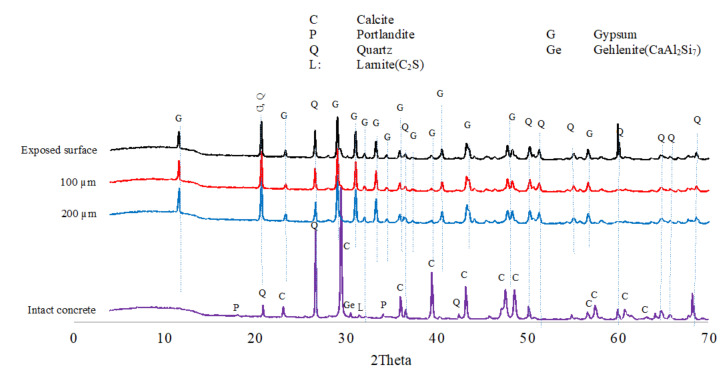
XRD analysis on the SRPC + FA concrete 25 months of exposure (Note: the above patterns include the aggregate phases (i.e., calcite and quartz), since they could not be excluded in the analyses).

The XRD analyses were carried out to identify the phases in the exposed concrete. Figure 11 shows XRD traces of the SRPC + FA concrete after 25 months of exposure. The analysis confirms the presence of portlandite, C_2_S, FeOOH, calcite, and quartz phases (from aggregates) in the intact concrete, as well as significant gypsum peaks at the exposed surface, and at 100 µm and 200 µm depth from the exposed surface. However, peaks corresponding to C-S-H were not observed, possibly reflecting the nanoscale crystalline structure of C-S-H because it is amorphous [77]. The XRD analysis of SRPC + FA + HC concrete (shown in the Appendix A, Figure A4) is similar to that of SRPC + FA concrete, implying that the iron-based additive does not participate chemically in hydration or biodeterioration processes.

**Figure 12:**
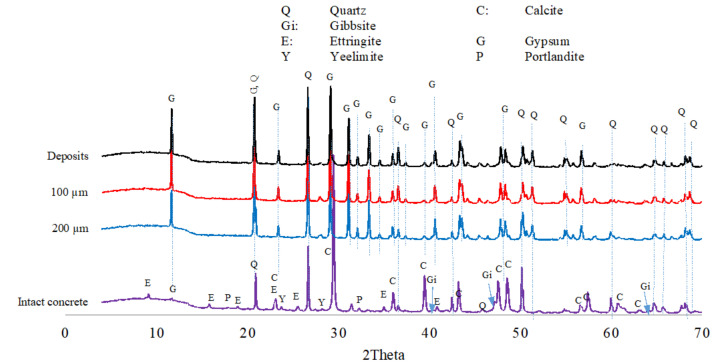
XRD analysis of CSA concrete after 25 months of exposure.

In the CSA concrete, ettringite, portlandite, gypsum, ye’elimite, and calcite and quartz from aggregates, were observed in the intact concrete, see Figure 12. At the exposed surface, a significant amount of gypsum is observed, thus supporting the transformation of ettringite to gypsum in the deterioration zone, as discussed in Section 3.1.4 and Section 3.1.5, as well as the dissolution of calcite aggregate, which provided calcium ions to react with sulphate ions from the acid to form gypsum. Ye’elimite shows the presence of anhydrous phases, which could promote further CSA hydration in the presence of absorbed moisture, as postulated above.

Generally, SRPC-based concrete was rich in calcium silicate hydrates and portlandite in the intact zone, and gypsum in the deterioration zone. CSA concrete contained ettringite, ye’elimite, and portlandite in the intact zone and gypsum in the deterioration zone. This confirms that SRPC concrete performance was due to C-S-H and portlandite decalcification from biogenic acid attack. In contrast, CSA performance was due more to ettringite formation and transformation to gypsum with sulphate penetration. More ettringite formation increases the neutralisation capacity of CSA, hence providing a superior performance.

### 3.2. Laboratory Conditions

#### 3.2.1. Microbial Activity

**Figure 13:**
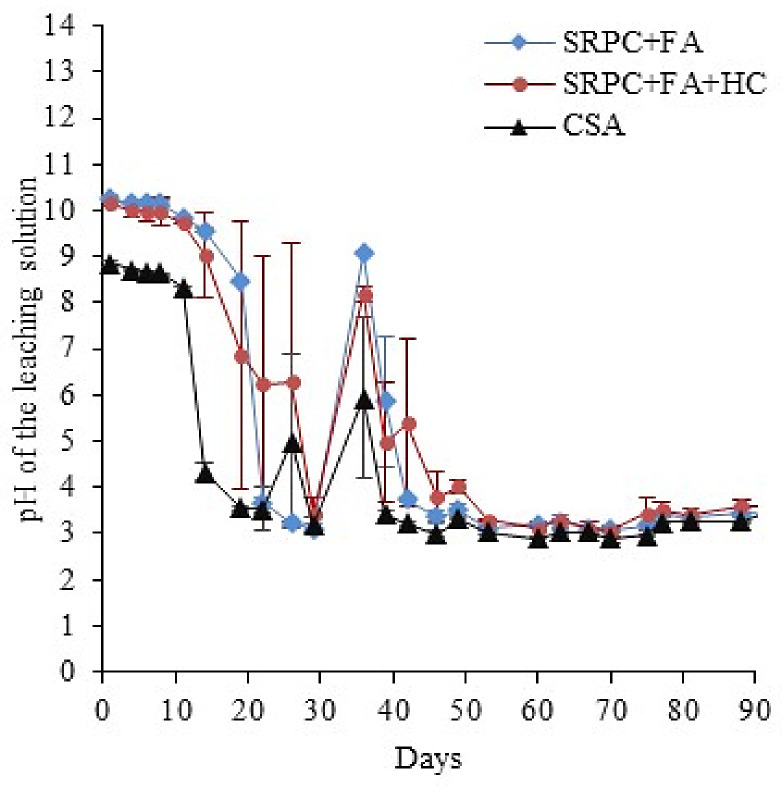
pH evolution during exposure to the BAC test for 3 months.

**Figure 14:**
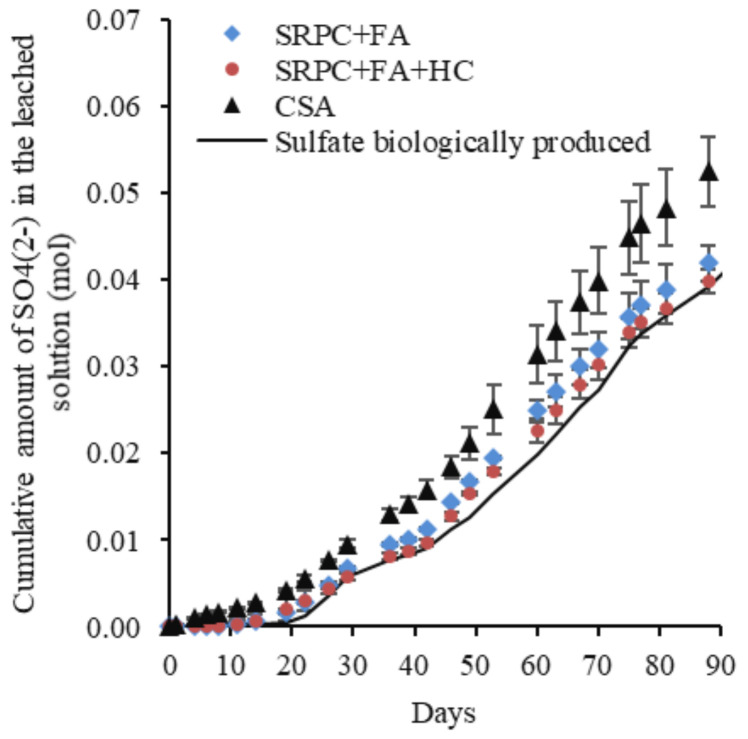
Cumulative measured sulphate concentrations in the leached solutions during exposure to the BAC test for 3 months.

As per Section 2.3, the development of microbial activity was monitored through the measurement of pH (Figure 13) and the concentrations of sulphate in the leached solutions (Figure 14). At day 35, the flow pump experienced a problem that interrupted the feeding solution for 24 h and led to surface drying of the exposed materials. As a result, the pH measurements at 36 days of exposure were relatively high. However, after 2 days, the pH of all specimens decreased, indicating that the biological activity was still efficient, leading to low and stable pH during the last 50 days.

Classically [54,64,65,66,78,79], at the beginning of the testing period (i.e., until 20 days), acidophilic SOBs were not active; thus, the pH of the leaching solution was that of the feeding solution, slightly alkalised by the contact with the cementitious material. Because the alkalinity of SRPC-based paste is higher than that of CSA [42], the pH of its leaching solution was higher than that of CSA (i.e., around 10 for SRPC-based, 9 for CSA). Afterwards, the SOB proliferated on the cement paste surface, producing sulfuric acid, leading to a progressive decrease in pH until reaching the stable value of around 3.5 for all three binder systems. In this way, the formation of an active sulfo-oxidising biomass was validated on all materials. Acidophilic conditions were established on the surface of the materials for several weeks under laboratory conditions. These results are largely mirrored in the field conditions, where very similar pHs are observed after 25 months of exposure (see the Appendix A, Figure A5).

The significantly larger error bars observed on the pH profile of the SRPC + FA + HC binder were associated with a technical problem in the feeding solution flow (clogging of its individual feeding tube). However, iron-based additives did not substantially impact the microbial activity, as the kinetics and the cumulative amount of sulphate were very similar for SRPC + FA and SRPC + FA + HC.

CSA cement paste, as already indicated, showed different pH and sulphate concentration behaviour in the leaching solution. Moreover, the acidification of the leached solution was faster than that of the SRPC-based paste, and the major drop in pH occurred at 14 days for CSA. The leaching solution from the CSA paste showed a higher amount of leached sulphate ions at high pH (before 14 days) than SRPC-based pastes, which was likely due to its higher initial sulphate content from dissolving sulphate-bearing phases, i.e., ettringite. From 14 days, the aggressiveness of the attacking acid increased, resulting in the dissolution of more sulphate-bearing phases. The estimation (Equation (1)) of the biologically produced sulphate confirmed the higher amount of the sulphate from the CSA materials compared to the SRPC + FA and SRPC + FA + HC. Thus, the following section further discusses the cumulative leached Ca and Al ions to distinguish the behaviour of these three binders.

#### 3.2.2. Evaluation of the Performance of Materials Using Cumulative Calcium and Aluminium in the Leached Solution

**Figure 15:**
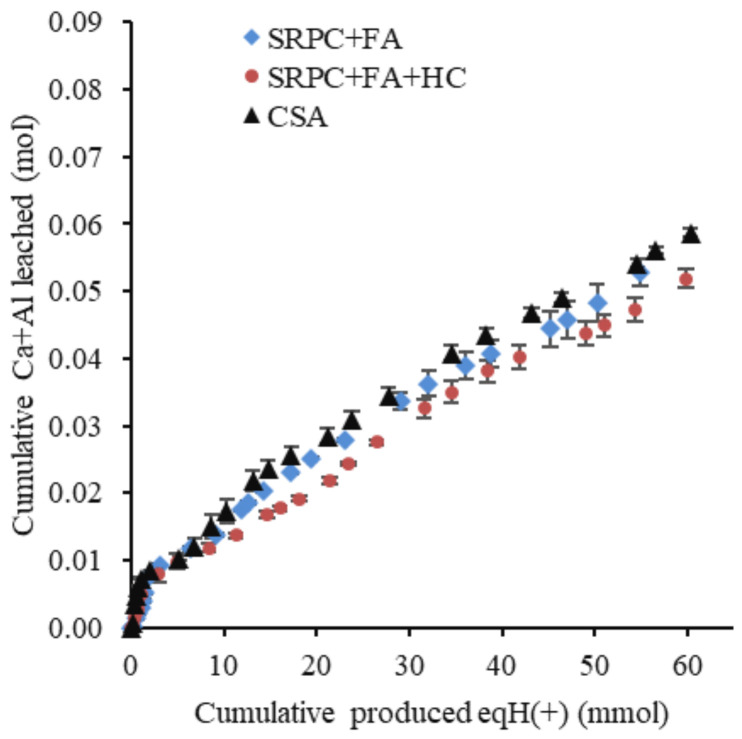
Cumulative Ca + Al leached as a function of the cumulative estimated biogenic acid in the leached solution for the cement pastes exposed to the BAC test.

**Figure 16:**
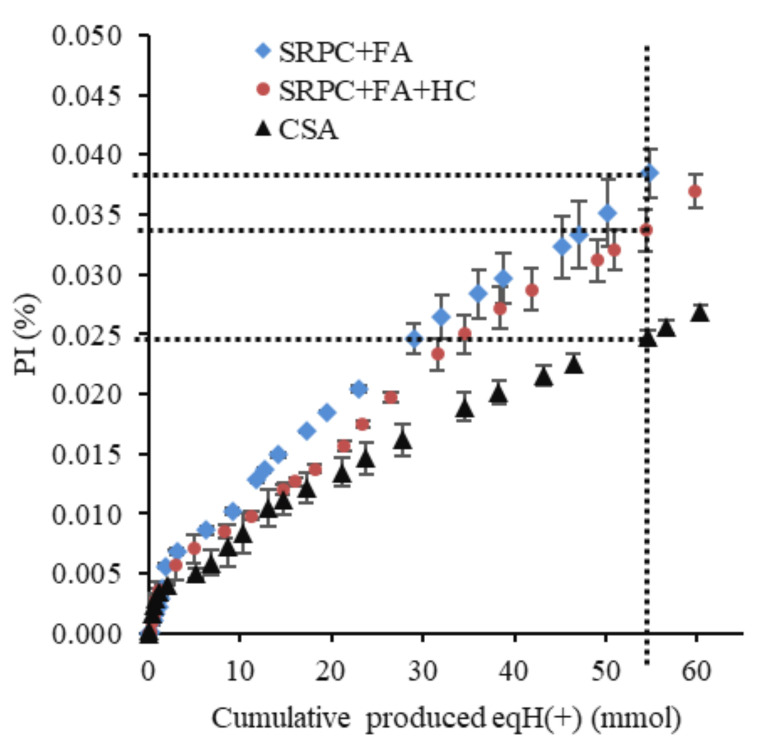
Standardised cumulative Ca + Al leached, expressed as performance indicator (PI) (For explanation of dotted lines, see paragraph below).

Figure 15 and Figure 16 present, respectively, the cumulative and the standardised cumulative (PI) leached calcium and aluminium (in moles) from the three cement pastes exposed to the BAC test against the cumulative estimated biologically produced acid (in moles).

The cumulative Ca and Al leached before standardisation could not definitively show performance differences among the three binders (Figure 15), indicating macroscopically a similar reactivity of the different binders with respect to acid. However, the materials do not have the same chemical composition, nor the same mineralogical composition. For this reason, standardised cumulative Ca and Al leached, expressed as performance indicators (PI), were calculated, accounting for the difference in the initial chemical and mineralogical composition of the cement paste. PI values (Figure 16) indicated that CSA ranked the lowest, followed by SRPC + FA + HC and SRPC + FA. For example, the PI values at 55 mmol of biologically produced acid were 2.45%, 3.40%, and 3.90% for the CSA, SRPC + FA + HC, and SRPC + FA, respectively. This implies that CSA experienced lower standardised Ca and Al leaching than SRPC-based material, hence better performance. The difference in performance between SRPC + FA + HC and SRPC + FA was further investigated using iron-leaching results (Section 4.1).

## 4. General Discussion

### 4.1. Performance of CSA and SRPC-Based Binder Systems Under Biogenic Acid Attack

The CSA cement used in this study was evaluated in terms of its performance compared to SRPC-based binders under biogenic acid attack. This cement has not been extensively studied under biogenic acid attack, particularly in ‘live’ sewer environments. This study showed that, over the period studied, CSA cement had superior performance to the SRPC-based binder system in terms of mass and thickness loss in the field, and standardised cumulative leached calcium and aluminium (PI) in the laboratory BAC test. While the work of Damion and Chaunsali [49] was not the same as the current study, the findings were similar, showing higher resistance of CSA-based compared to full PC-based matrices in terms of normalised residual flexural strengths after one year of field exposure in a sewer.

In the field study, CSA showed significant mass gain up to 18 months of exposure, followed by minor mass loss after 25 months. The mass gain is likely related to ongoing CSA hydration due to moisture absorption and the formation of additional hydration products, mainly ettringite, as it is the dominant hydration product of CSA cement. Mass gain also occurs initially due to the formation of deterioration products, specifically either ettringite or gypsum, depending on the pH in the concrete microstructure.

The literature indicates that CSA cement requires w/b ratios greater than 0.40 for full hydration [19,73,80]. Since a 0.40 w/b was used in this study, CSA concrete had an anhydrous phase, mainly ye’elimite, which could be expected to undergo further hydration in the presence of moisture to produce more ettringite. On the other hand, with subsequent sulphate penetration, additional ettringite was formed in the deeper sections of the concrete microstructure at a pH greater than 10.7 [13,81,82].

At pH under 10.7, ettringite dissolves to Ca^2+^, Al(OH)_4_^−^, and SO_4_^2−^ ions, see Equation (5) [83]. Under a sufficient concentration of SO_4_^2−^ and Ca^2+^, gypsum precipitates, while providing more OH^−^ for neutralising sulfuric acid. Therefore, if the neutralisation capacity of all three binder systems is expressed as an OH^−^ equivalent based on the material composition, i.e., CaO, Al_2_O_3_, Fe_2_O_3_, MgO and SO_3_ for the binder and reactive aggregates, as shown in Table 1, CSA concrete has the highest neutralisation capacity of 37,943 eqmolOH^−^/m^3^, followed by 37,634 eqmolOH^−^/m^3^ for SRPC + FA + HC and 36,662 eqmolOH^−^/m^3^ for SRPC + FA concrete. A similar trend is observed in the cement paste, where the values are 10,402 eqmolOH^−^/m^3^ for CSA, 10,093 eqmolOH^−^/m^3^ for SRPC + FA + HC and 9121 eqmolOH^−^/m^3^ for SRPC + FA. The higher neutralisation capacity in concrete compared to cement paste is attributed to calcite aggregates. This higher neutralisation capacity of CSA compared to that of PC-based matrices was also highlighted experimentally by Damion et al. [53].

3CaO.Al_2_O_3_.3CaSO_4_.32H_2_O → 6Ca^2+^ + 2Al(OH)_4_^−^ + 3SO_4_^2−^ + 4OH^−^ + 26H_2_O (5)

**Figure A2:**
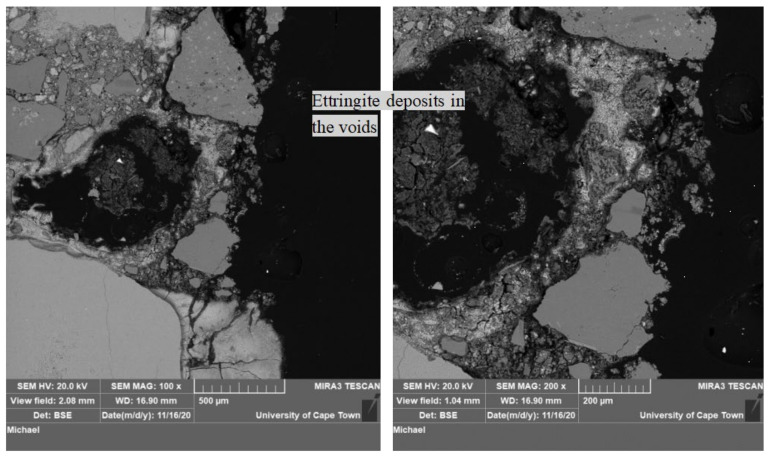
Formation of ettringite in the CSA concrete voids.

**Figure A3:**
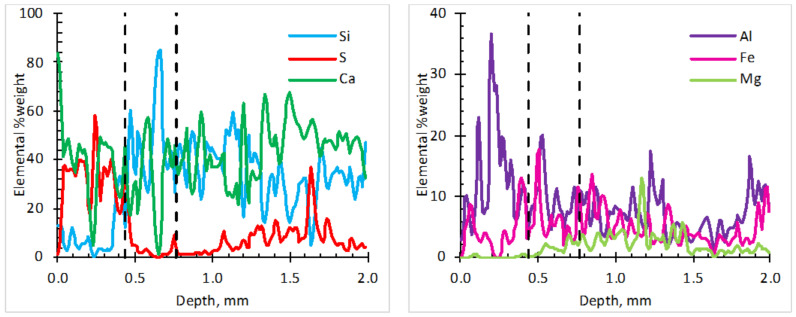
SEM-EDS elemental analysis on a 2 mm depth of SRPC + FA + HC concrete samples from the exposed surface; calcium (Ca); silicon (Si); sulphur (S); aluminium (Al); iron (Fe); and magnesium (Mg). Three zones, i.e., deterioration, transition, and intact zone, are shown inwards from the left-hand axis.

**Figure A4:**
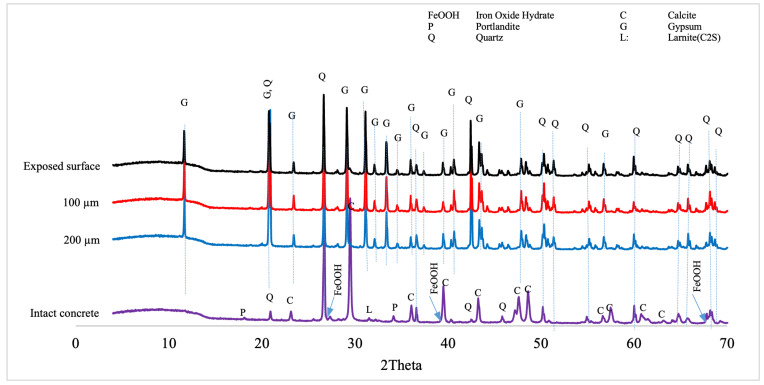
XRD analysis of SRPC + FA + HC concrete after 9.6 months of exposure.

**Figure A5:**
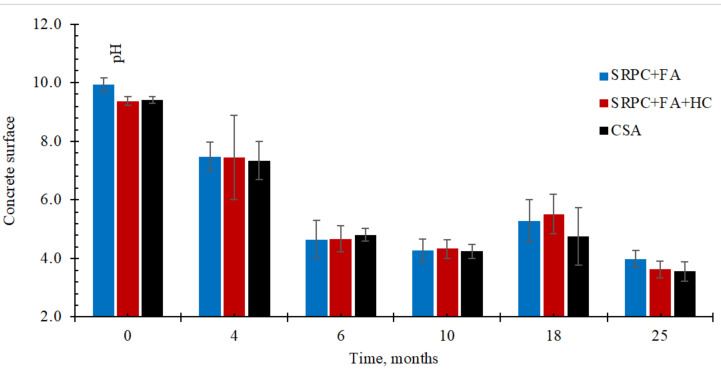
pH measurements on the surface of concrete specimens exposed at NAS M19.

However, due to the expansive nature of ettringite and its instability at pH less than 10.7, it is postulated to result in internal cracking and loss of strength under acid attack [39]. As indicated earlier, porosity and voids in the CSA concrete microstructure were postulated to compensate for the expansive effect of ettringite formation (see Figure A2). Later, visible cracking was observed in the CSA epoxy coating surfaces. XRD analysis showed the presence of ettringite in the intact zone, while gypsum peaks were observed in the deteriorated zone.

The results from the laboratory testing confirmed the field observations, exhibiting similar performance ranking between the three binders. For example, CSA experiences lower standardised Ca and Al leaching (lower PI) than SRPC-based material, giving better performance.

The results from both field and lab tests also demonstrate the different stages of the biogenic attack (NSOB and then ASOB)—see Figure 6, Figure 7, and Figure 13. For the consideration of deterioration rates, the ‘steady state’ of acid attack is required, see the linear portions of the plots presented. The ‘incubation stage’ (related to the commencement of the ASOB growth) is clearly visible in the field results in Figure 6—approximately 18 months for the CSA and 5 months for the SRPC systems. However, this is less visible and reversed in the BAC test, Figure 13 (25 days for the SRPC, and 12 days for the CSA). The incubation period in the field may appear longer than for PC, as the high porosity of the CSA may enable the growth of secondary products without any detectable mass losses and deteriorated depths for a significant duration. In contrast, in the BAC test, the tests were carried out on the paste with no accentuated porosity compared to the SRPC materials. The deterioration phenomena correspond to surface dissolution attacks, where the diffusion phenomenon plays only a secondary role, limiting the influence of secondary precipitations in the deeper zones of the materials. Finally, in the BAC test, the incubation phenomenon is related to the intermediate hydroxide potential of the tested material, which will delay the acidophilic SOB activity [52,84] and which is higher for SRPC than for CSA, with no impact on the ranking of the materials.

Further, field- and lab-based results are notable for their consistent comparisons between SRPC and CSA binders. The surface deterioration was similar in the two binder types, but mass losses were lower for CSA than for SRPC-based concrete, while leaching of Ca+Al was about 28% less at 55 days compared to the SRPC+HC and 37% less than SRPC. The reasons for the improved performance of CSA over SRPC relate to its higher neutralisation capacity thereby consuming more acid per unit of binder, the chemical nature of the materials and their acid-base reactions, and the better stability of secondary phases such as gibbsite (AH_3_) showing superior performance to Al-Si gel.

In the following section, the slight difference in performance between the two SRPCs—one with and one without a surface hardener (HC)—is examined, mainly for the influence of the hardener.

### 4.2. Role of Iron-Based Additive (HC) in Concrete Under Biogenic Acid Attack

This study incorporated an iron-based additive (HC) into one SRPC-FA-based concrete to study its role in concrete performance under biogenic acid attack. This concrete showed a smaller mass loss and thickness loss over the 25-month period of measurement, about 2.3% and 3.7 mm, respectively, than the equivalent concrete without the additive, with corresponding value of 3.2% and 5.6 mm, respectively (see Figure 7). However, its definitive role was not clearly identified.

Under SEM-BSE imaging, the additive particles appeared not to participate in either hydration or dissolution reactions. Under SEM-EDS analysis, iron peaks in the concrete without iron-based additive were observed only in the transition zone, while smaller iron peaks were observed across the depth in the concrete with the additive, reflecting the presence of the additive throughout the mass. XRD analysis showed that the iron-based additive had not influenced the hydrated material.

**Figure 17:**
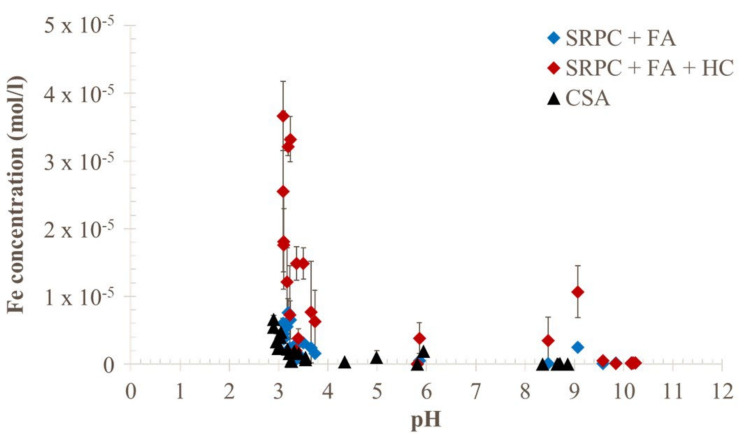
Iron concentrations in the leached solutions of the three exposed materials as a function of the pH of the leached solutions.

**Figure A6:**
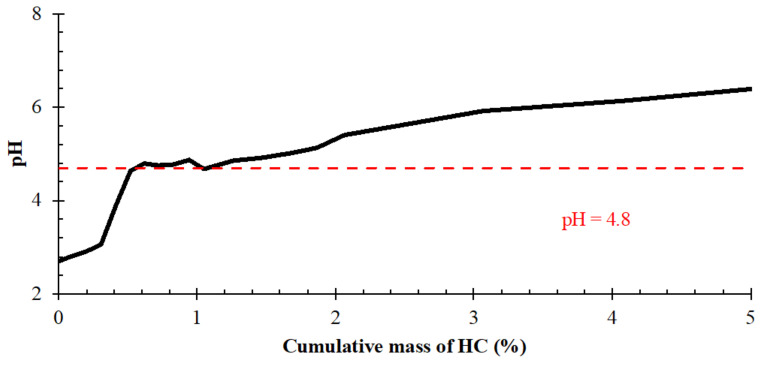
pH evolution of the solution as a function of the cumulated added mass of iron-based additive (HC).

The impact of incorporating an iron-based additive into the cementitious matrix was further investigated by monitoring the iron concentrations in the leached solutions for the three materials exposed to the BAC test as a function of pH to evaluate its reactivity under biogenic sulfuric acid attack (Figure 17). At pH > 10, no iron was found in the leached solutions for all cement pastes. Low iron concentration was detected for 4 < pH < 10, particularly for SRPC + FA + HC. Finally, for pH < 4, the iron concentrations increased substantially for all materials, but more particularly for SRPC + FA + HC, sensibly due to the presence of the iron-based additive. Thus, for further understanding, the intrinsic chemical acid resistance of the iron-based additive was evaluated by exposing anhydrous grains to a sulfuric acid solution at an initial pH of 2.7. The results in Figure A6 showed a slow dissolution of the iron-based additive occurring at a pH > 5, and a very rapid dissolution below. This is consistent with the significant increase in iron concentration at pH < 4 for the SRPC + FA + HC mix (Figure 17), implying the instability of additive iron in an acidic environment. However, this instability is not apparent in the field results, possibly due to its low content dispersed in the concrete microstructure. Hence, the SEM-EDS analysis of the hydrated cement pastes of SRPC with and without HC were conducted to investigate the reactivity of iron-based additives in the hydrated cement pastes (Figure 17).

**Figure 18:**
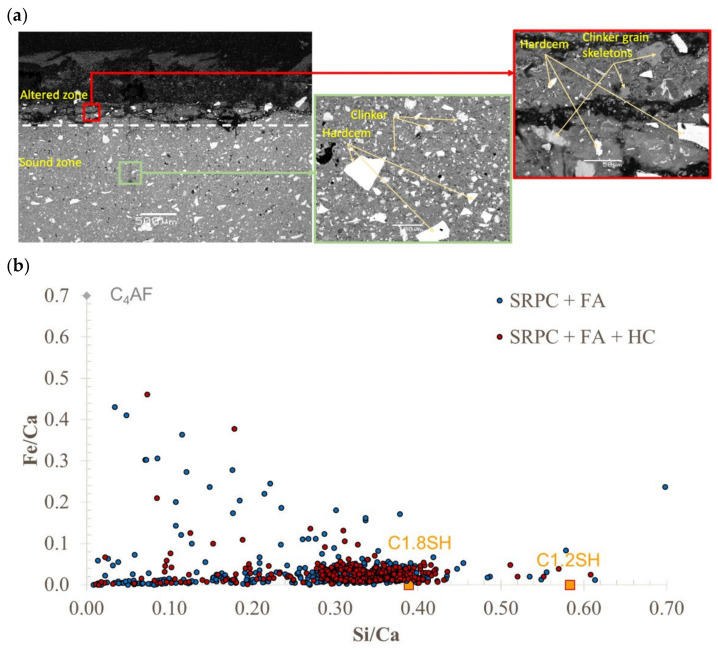
(**a**) BSE-SEM observation of cross sections of SRPC + FA + HC cement paste exposed to the BAC test, surface exposed to the biofilm on the top. Overview of altered and sound zones of the specimen (**left**), higher magnification image of the paste in the sound (**middle**) and in the altered zone (**right**) showing the HC and clinker grains, and the surrounding hydrated paste. (**b**) Fe/Ca ratio as a function of Si/Ca from EDS analysis of the hydrated cement paste for SRPC + FA and SRPC + FA + HC in the sound zone.

Figure 18 presents BSE-SEM observation of cross sections of SRPC + FA + HC cement paste exposed to the BAC test, showing the iron-based additive and clinker grains, the surrounding hydrated paste in the altered and sound zones, and the Fe/Ca atomic ratio as a function of the Si/Ca ratio of the hydrated cement paste for SRPC + FA and SRPC + FA + HC materials from SEM analyses in the sound zone. The results show that the ratio of Fe/Ca was low in both materials, generally less than 0.1. In addition, the hydrated cement pastes of SRPC + FA + HC did not show any difference in terms of iron enrichment compared to SRPC + FA. This indicates that the iron-based additive did not participate in the hydration of the matrix, as shown by the very sharp contrast between the HC grains themselves and the surrounding paste, suggesting no incorporation of highly dense iron-based particles in the hydrated phases (Figure 17). This is consistent with the observations on the concrete field experiments, see Section 3.1. SEM observations in Figure 18a also confirm that the iron-based additive grains are completely dissolved in the outer part of the altered zone.

From another point of view, the dissolution of iron-containing phases in cementitious materials increases the acid neutralisation capacity since OH- ions are released and react with the acid, causing pH buffering. By comparing the iron-based additive to calcium-bearing phases (such as CH), the additive (and ferrous phases in general) can have a positive impact since they have higher chemical stability to acid attack, down to a pH of around 5.

Overall, this additive was beneficial in improving the resistance of the concrete to surface loss. This effect appears to be largely physical by providing better surface hardness, which resists the removal of deteriorated material (better behaviour was obtained for concrete with iron-based additive in a previous field study, data not published). Based on these observations, it could be expected that this effect would be maintained over a more extended period and would be beneficial in the tidal zone of the sewer, where hydraulic erosion contributes to the deterioration.

## 5. Conclusions

This work aimed to evaluate the performance of CSA and SRPC + FA binder systems in a ‘live’ field sewer environment, and to deepen the understanding of their deterioration mechanisms using the simulated laboratory sewer test condition. A secondary objective was to explore the role of an iron-based additive in possibly improving the performance of SRPC + FA concrete under biogenic acid attack.

The following points were extracted as the main findings of this study.

(i) CSA cement had superior performance in the live sewer environment and also in laboratory conditions, compared with SRPC-based binder systems, when assessed using performance indicators of mass loss, thickness loss, and standardised cumulative leached calcium and aluminium in the laboratory test.
(ii) Concrete specimens subjected to a live sewer environment initially experienced mass gain due to moisture absorption before they experienced mass loss due to deterioration. For concrete with CSA cement and a low water/binder ratio, sewer moisture likely activates hydration of the remaining un-hydrated cement within the microstructure, consequently causing mass gain, compensating for the mass loss due to thickness loss.
(iii) The superior performance of CSA is associated with further ettringite formation due to sulphate penetration, which, at pH <10.7, dissolves to provide more CSA neutralisation capacity to acid penetration than in the SRPC-based system.
(iv) The iron-based additive used in this study reduced the mass loss and thickness loss of SRPC-based concrete to a minor extent, due to its influence in improving concrete integrity by providing resistance against abrasion and erosion (i.e., sewer hydraulic erosion), but not due to its chemical influence, since it participates in neither hydration nor dissolution reactions. Questions remain as to whether this effect (a) is maintained over an extended period, and (b) whether it would be beneficial in the sewer tidal zone, where hydraulic erosion is a significant form of deterioration.

In summary, this study found that CSA cement may represent a suitable alternative binder for concrete sewer construction, but the use of a surface hardener would likely have limited benefits under abrasive conditions.

## Figures and Tables

**Table 1:** Chemical composition (in wt. %) of the cements used in this study.

Reference	CaO	SiO_2_	Al_2_O_3_	Fe_2_O_3_	MgO	SO_3_	TiO_2_	Others	LOI
SRPC	67.95	21.45	3.14	2.17	0.95	1.85	0.14	0.68	1.63
CSA *	41.75	4.70	30.37	1.53	0.62	15.71	1.26	0.35	2.73
HC ^#^	17.05	33.61	5.10	40.69	0.93	2.12	0.32	2.55	−4.12
FA	4.32	53.72	32.94	3.23	1.07	0.00	1.71	1.94	1.03
Anhydrite	Ye’elimite	Gehlenite	Perovskite	Calcite	Magnetite	Mayenite	C_2_Sα	Total	
29.50	33.52	18.52	5.08	8.04	1.45	1.01	2.73	100	

^#^ Negative LO1 indicates the loss of combined water and not gas during ignition; * Mineralogical phase composition of anhydrous CSA, wt. %.

**Table 2:** Concrete mix proportions for site exposure—kg/m^3^.

Mix Designation		SRPC + FA	SRPC + FA + HC	CSA
Binder Systems		SRPC 80% + FA 20%	SRPC 80% + FA 20% + HC (11.4%)	100% CSA
w/b		0.4		
**Constituents**		**kg/m^3^**		
Binder		280	280	350
Fly Ash (FA)		70	70	0
Iron-based additive (HC) ^a^		0	40	0
Water		133	133	133
Fine aggregates	0/1 mm Siliceous	520	520	520
	1.6/3 mm Calcite	520	520	520
	3/6 mm Calcite	343	343	343
Coarse aggregates	6/11 mm Calcite	516	516	516
BASF-Glenium 7700 (% by mass, binder)		0.64	0.64	1.08
% Total binder		16	16	16
Theoretical density of concrete		2383	2422 ^a^	2383
28-day compressive strength (MPa)		44.8 ± 11.0	43.8 ± 8.1	40.3 ± 3.9
28-day saturated density (kg/m^3^)		2379	2339	2320
OPI (log)		10.7	10.6	10.7
WSI mm/h^0.5^		5.4	6.0	7.1
Water-penetrable porosity %		8.1	8.4	6.9

^a^ HC was an added constituent to this mix. The proportions for this mix are reported as shown since the iron-based additive represented a minimal volumetric component.

## Data Availability

The original contributions presented in this study are included in the article. Further inquiries can be directed to the corresponding author.
